# Incised Wound of Intestine—Recovery

**Published:** 1881-11-20

**Authors:** W. A. Newborn

**Affiliations:** Oakland, Tenn.


### INCISED WOUND OF INTESTINE—RECOVERY.

BY W. A. NEWBORN, M. D., OAKLAND, TENN.

On July 25th I was called in connection with Dr. J. McCulley, to see a negro boy 15 years of age, who had been partially disemboweled by his antagonist. We did not reach the boy until several hours after the accident. Hence we found the protruded mass so swollen that we were compelled to enlarge the wound to accomplish its reduction.

The wound was situated in the left lumbar region, internal to the anterior superior spinous process of ilium two inches, and extending toward the median line two inches. The colon was wounded in three places, two punctures and one incision three-quarter inch in length; on any exertion of the patient the fœcal matter would ooze from the largest wound in a stream as large as one's little finger; but as the bowel protruded, I hardly think any of the fœcal matter escaped into the peritoneal cavity, at least no symptoms followed to indicate this.

To close the wounds in the colon required four stitches; catgut not being at hand we used silk sutures. After replacing the mass we allowed the drainage for a few minutes, then closed the external wound by five stitches, using the interrupted suture. We used Lister's antiseptic method throughout. Placed the patient on quarter grain morphia every six hours, to relieve any pain and reduce peristalsis. On the sixth day his symptoms growing worse, gave enema, which caused a discharge of scybala, followed by marked relief to tympanites.

On the ninth day his symptoms became alarming, tympanites excessive, pulse 138, respiration 26 to 30, temperature 101½°. We, after some hesitation, gave a dose of castor oil, and waited with fear for the result. It caused several copious and fœtid evacuations without much pain and with great relief to tympanites, pulse fell to 120; respiration 22; temperature 100⅓°. He made a gradual improvement from this; had occasional fever without any chill. After this there was a tendency to diarrhœa, so we changed from quarter grain morphia to one grain powdered opium every five hours. This controlled the bowels.

Our principal treatment throughout was to meet the indications. We kept ice bags on the wound for the first ten days—used bismuth subnit and pepsin to allay irritability of the stomach—used turpentine emulsion per ingesta, and turpentine liniment to bowels to allay tympanites—quinine in tonic doses. The sutures in the external wound became irritant, and as the wound was healing from the bottom, we removed the sutures and applied adhesive strips so as to give support to the wound. Then sprinkled into the wound iodoform and salicylic acid. From this on the wound did splendidly, granulating from the bottom. Convalescence being established we then used tr. iron 10 gtt. three times per day. His diet was Tanner's plan mostly, but gave liquid food enough to support him.

After three weeks we discharged the patient; and gave him instructions to be careful in his diet. I regard his case as one of great interest and one in which prognosis is generally grave.—*Nashville Jour. of Medicine and Surgery.*

---

The six healthiest cities in the United States, as measured by the most recent authentic reports, were in the order named; Utica, Dayton, New Haven, Portland, San Francisco, and Lawrence. The six unhealthiest were Charleston, Memphis, Cleveland, Chicago, Hudson County, N. J., and Lynn. The six unhealthiest in the world were St. Petersburg, Charleston, Malaga, Alexandria, Warsaw and Blue-Pesth.—*N. Y. Medical Record.*

